# Self-management support program delivered in the sub-acute phase after traumatic injury—study protocol for a pragmatic randomized controlled trial

**DOI:** 10.1186/s13063-024-08492-0

**Published:** 2024-09-30

**Authors:** Mari S. Rasmussen, Nada Andelic, Joanna Nordhagen Selj, Vilde Marie Danielsen, Marianne Løvstad, Emilie Isager Howe, Torgeir Hellstrøm, Helene L. Soberg, Cathrine Brunborg, Eline Aas, Håkon Moksnes, Unni Sveen, Christine Gaarder, Pål Aksel Næss, Eirik Helseth, Olav Røise, Mads Aarhus, Hege Prag Øra, John Andreas Bjørneboe, Silje Fure, Cecilie Røe, Christoph Schäfer, Paul B. Perrin, Juan Lu, Marie Elf, Hilde Margrethe Dahl, Fiona Jones, Jennie Ponsford, Linda Narvestad, Solveig L. Hauger

**Affiliations:** 1 Department of Physical Medicine and Rehabilitation, Oslo University Hospital, Oslo, Norway; 2 Faculty of Health Sciences, Oslo Metropolitan University, Oslo, Norway; 3 Research Centre for Habilitation and Rehabilitation Models & Services (CHARM), Faculty of Medicine, Institute of Health and Society, University of Oslo, Oslo, Norway; 4 Institute of Clinical Medicine, Faculty of Medicine, University of Oslo, Oslo, Norway; 5 Department of Research and Innovation, Sunnaas Rehabilitation Hospital, Nesoddtangen, Norway; 6 Department of Psychology, Faculty of Social Sciences, University of Oslo, Oslo, Norway; 7 Oslo Centre for Biostatistics and Epidemiology, Research Support Services, Oslo University Hospital, Oslo, Norway; 8 Department of Health Management and Health Economics, Institute of Health and Society, Faculty of Medicine, University of Oslo, Oslo, Norway; 9 Division for Health Services, Norwegian Institute of Public Health, Oslo, Norway; 10 Department of Traumatology, Oslo University Hospital, Oslo, Norway; 11 Division of Orthopaedic Surgery, Department of Neurosurgery, Oslo University Hospital, Oslo, Norway; 12 Department of Neurosurgery, Oslo University Hospital, Oslo, Norway; 13 Department of Rehabilitation, University Hospital of North Norway, Tromsø, Norway; 14 Department of Clinical Medicine, University Hospital of North Norway, Faculty of Health Sciences, Tromsø, Norway; 15 Departments of Psychology and Physical Medicine and Rehabilitation, Virginia Commonwealth University, Richmond, VA USA; 16 Department of Family Medicine and Population Health, Division of Epidemiology, Virginia Commonwealth University, Richmond, VA USA; 17 Department of Nursing 2, School of Health and Welfare, Dalarna University, Falun, Sweden; 18 Department of Clinical Neurosciences for Children, Section for Child Neurology, Oslo University Hospital, Oslo, Norway; 19 Population Health Research Institute, St George’s, University of London, London, England UK; 20 Bridges Self-Management, London, England UK; 21 Monash-Epworth Rehabilitation Research Centre, Epworth Healthcare, Richmond, Australia; 22 Monash Institute of Cognitive and Clinical Neuroscience, School of Psychological Sciences, Monash University, Melbourne, Australia; 23 Department of Subjects and Development, Oslo Municipality, Oslo, Norway

## Abstract

**Background:**

Traumatic injuries, defined as physical injuries with sudden onset, are a major cause of distress and disability, with far-reaching societal consequences. A significant proportion of trauma survivors report persistent symptoms and difficulties after the injury, and studies show unmet health care needs. Self-management programs delivered in the sub-acute phase after traumatic injuries are scarcely evaluated. The aim of the present study is to evaluate the effectiveness of a self-management program (SEMPO), delivered 3–4 months after moderate-to-severe traumatic injury.

**Methods:**

This study protocol describes a pragmatic randomized controlled trial (RCT) with two classical RCT arms (intervention and control) and an explorative self-selection arm. 220 patients will be recruited from Oslo University Hospital, the largest Trauma Referral Centre in Norway. Patients aged 18–72 years residing in the south-east region of Norway, admitted to the Trauma Centre directly or within 72 h after having sustained a moderate to severe traumatic injury, defined as a New Injury Severity Score > 9, having at least 2 days hospital stay, and reporting injury-related symptoms and impairment at discharge from the acute hospital will be included. Patients will be randomly assigned to either a classical RCT randomization arm (intervention or control arm) or to a self-selection arm. In the randomization arm, participants are further randomized into intervention or control group. Participants allocated to the self-selection arm will choose to partake either in the intervention or control arm. The primary outcome is the level of self-efficacy in trauma coping assessed 6 months after completion of the intervention, with a similar time point for the control group. Secondary outcomes include symptom burden, physical functioning and disability, return to work and health care utilization, health-related quality of life, and communication competency. In addition, patients will be asked to nominate one domain-related measurement as their preferred outcome measure.

**Discussion:**

This RCT will determine the effect of a self-management program tailored to patients with moderate to severe physical trauma, and the self-selection arm incorporates the potential influence of patient treatment preferences on intervention results. If the intervention proves effective, cost-effectiveness and cost-utility analyses will be performed and thereby provide important information for clinicians and policy makers.

**Trial registration:**

The study is registered in Clinical Trials with the identifier: NCT06305819. Registered on March 05, 2004.

**Supplementary Information:**

The online version contains supplementary material available at 10.1186/s13063-024-08492-0.

## Introduction

### Background and rationale

Traumatic injuries, defined as physical injuries with sudden onset, are a major cause of distress and disability, with far-reaching societal consequences [1]. Traumatic injuries may cause difficulties in physical, cognitive, emotional, and behavioral functioning. A significant proportion report problems in daily life activities, impaired physical and mental health, and reduced quality of life years after the injury [2–4]. This may subsequently limit participation in work, studies, leisure activities, and family life.

Results from a Norwegian multi-center follow-up study on rehabilitation service provision after trauma showed that approximately 50% had a persistent disability at 12 months post-injury, as assessed with the Global Outcome Scale Extended (GOSE) [5]. Patients report prolonged problems with physical, cognitive, and emotional functioning, as well as pain, fatigue, sleep impairment, and reduced participation. Furthermore, preliminary results from the follow-up study demonstrated that 59% and 46% had unmet needs for health care and rehabilitation services 6 and 12 months after the injury. A previously published study on persons with moderate to severe traumatic injury demonstrated that persons who had a pre-injury comorbidity, a higher number of injuries, and higher estimated rehabilitation needs were at risk of poorer functional outcomes 1 year after the injury [5].

Also, persistent disability has emerged as a potential mediating factor for the delayed onset of psychological issues [6, 7]. Despite the fact that most recovery occurs within the first 6 months after injury, the frequency of post-traumatic stress symptoms has shown to remain high [8], and commonly with a delayed onset following traumatic injury [9]. Taken together, interventions aiming to reduce psychological distress in the context of disability should be included in all phases of rehabilitation after traumatic injuries, particularly in the sub-acute phase to support resilient recovery trajectories. Although the need to improve rehabilitation services and provision, including shared decision-making and self-management approaches has been acknowledged [10], rehabilitation following traumatic injuries is still under-prioritized, and self-management approaches are lacking.

The concept of self-management support (SMS) refers to the systematic provision of education and supportive interventions to increase patients’ skills and confidence in managing their health problems, which is in line with the principles of user involvement and patient-centered health services [11]. Self-management as a health concept builds on the theory of self-efficacy developed by Bandura [12] and relates to one’s confidence in the capability to organize and execute the courses of actions required to produce given attainments related to health [11]. Self-management programs aim to enhance self-efficacy by building skills in decision-making, use of available resources, goal setting, and action planning [13]. Self-management also builds on theories of problem-solving, understood as the capacity for effective and adaptive ways of coping with problematic life situations [14].

To date, self-management programs have primarily been directed toward persons with chronic conditions such as asthma, arthritis, diabetes, mental illness, pain, cancer, and AIDS [15]. Studies of self-management interventions have shown significant improvements in health behavior, health status, self-efficacy, fatigue, health-related quality of life, and health care utilization [15–18]. Surprisingly, few studies have investigated the effectiveness of such programs in the early phases of injury or illness, and knowledge concerning the effectiveness of SMS programs for those with traumatic injuries is lacking. Of note, a manualized program developed for chronic disease covers topics that overlap with problem areas reported by the trauma population, such as physical, cognitive, and emotional functioning, fatigue and sleep management; and the use of community resources [15]. One study applied a self-management intervention tailored to the early phase after stroke. The results showed positive changes in self-efficacy, functional activity, social integration, and quality of life [17]. Considering the long-term symptom burden and disabilities following trauma, in addition to the uneven distribution of rehabilitation services, there is a need to investigate the effect of self-management support on patients with persistent injury-related symptoms and difficulties in the sub-acute phase. A recent meta-analytic review by Carlisle et al. found that the choice-based behavioral and mood intervention may enhance participant retention and adherence and may be beneficial to the outcome as well [19]. In order to improve the evidence- base they recommended to consider the provision of choice when designing research and interventions.

It is believed that giving patients the choice to select the treatment or not may improve patient satisfaction and preferences. This may be essential to good clinical practice because the patient’s cooperation and satisfaction reflect the degree to which medical intervention fulfills his or her choices, values, and needs.

### Objectives

The main objective of this study is to determine the effectiveness of a self-management program aiming to support recovery trajectories and prevent chronic problems in patients with moderate to severe traumatic injuries. A self-management support program, SEMPO, was developed for patients in the sub-acute phase of moderate to severe traumatic injury.

The SEMPO is a group-based intervention that includes eight weekly sessions focusing on a specific topic, and it is theoretically based on self-management principles and established rehabilitation strategies. The intervention comprises several ingredients, including psychoeducation, guided skills mastery, learning and practicing helpful compensatory strategies and problem-solving techniques, as well as sharing coping experiences.

We hypothesize that compared to the control group, SEMPO will result in:

- Increased self-efficacy in managing health problems
- Improved health, reduced disability, and use of fewer health care resources
- Improvement in target problem areas and lower burden of trauma-related problems
- Evidence on the relative effectiveness and cost-effectiveness of SEMPO compared to standard services

We further anticipate that:

- Self-selected partaking in the intervention will enhance self-efficacy and level of function of the self-nominated outcome, as well as maximize adherence and reduce potential attrition bias
- Implementation fidelity will be high and the intervention will be deemed acceptable by participants and therapists

### Trial design

The proposed study is a pragmatic parallel-group randomized controlled trial (RCT) with a mixed method design. The study design includes the classical RCT design with randomization to intervention and control arm. In addition, patients could be randomized to a self-selection arm where they can choose to be allocated to either the intervention or control arm. This is considered as an exploratory part of the study to explore whether the patients’ treatment preferences will maximize adherence, reduce potential attrition bias, and influence the intervention results [20]. Hence, this innovative pragmatic RCT seeks to align the study design to patient-centered treatment, where the influence of patient treatment preferences on intervention effect and adherence will be explored.

Patients who consent to participate will undergo a baseline assessment (T1) before being randomized to either the randomization (RA) arm or self-selection (SA) arm. Follow-up assessments will take place after the completion of the 8-week SEMPO program (T2) and at 3 (T3) and 6 months (T4) after completion of the intervention, with similar follow-up time points for the control group. A feasibility study will be conducted before the full-scale RCT. Additionally, a process evaluation will be performed. This mixed-method design is in line with an updated recommendation by the British Medical Research Council [21].

## Methods: patients, interventions, and outcomes

### Study setting

The study will be conducted at Oslo University Hospital (OUH), Dept. of Physical Medicine and Rehabilitation. OUH is the trauma referral center for the South-eastern region of Norway and has a population base of more than half of the Norwegian population. The feasibility study was conducted from autumn 2023 until January 2024, while the recruitment of patients for the RCT started in January 2024 and will continue until the required sample size has been reached. All baseline assessments will be performed at OUH, interventions either at OUH or by telehealth, while follow-ups will be performed either at OUH or by phone.

### Eligibility criteria

The study population consists of persons aged 18–72 years residing in the southeast region of Norway, who are admitted to OUH directly or within 72 h after having sustained a moderate to severe traumatic injury, defined as a New Injury Severity Score (NISS) > 9 [22], who have at least a 2-day hospital stay, and report injury-related symptoms, functional impairment, and/or difficulties with daily life activities at discharge from the acute hospital stay. Those who consent to participation and are allocated or by self-selection choose to be in the treatment group, will receive SEMPO 3–4 months after the injury.

Exclusion criteria are cognitive functioning corresponding to a Mini-Mental Health Status [23] score < 20 points, severe psychiatric disease or drug/alcohol dependence requiring treatment, complete spinal cord injury or isolated abdominal or thoracic injuries, and insufficient command of Norwegian. The reason for excluding persons with complete spinal cord injuries is that these patients undergo comprehensive long-term rehabilitation of which we will not interfere. The reason for excluding persons with isolated abdominal or thoracic injuries is that a previous Norwegian longitudinal follow-up study demonstrated that these patients seldom have rehabilitation needs [24]. Severe psychiatric disease or drug/alcohol dependence requiring treatment will be excluded due to likely interference with participation and treatment outcomes (Table 1).

**Table 1:** Standard Protocol Items: Recommendations for Interventional Trials (SPIRIT)

		**Study period**					
	**Enrolment**	**Baseline assessment**	**Allocation**	**Intervention**	**Outcome assessment**		
**Timepoint**		***t***_**1**_			***t***_**2**_	***t***_**3**_	***t***_**4**_
**Enrolment:**	X						
**Eligibility screen**							
**Informed consent**	X						
**Allocation**			X				
**Interventions:**				X			
***A: Self-management program (SEMPO)***							
***B: Treatment as usual***				X			
**Assessments:**		X			X	X	X
***Primary outcome assessment* (***self-efficacy)*							
***Secondary self-selected outcome assessment**** (symptom burden, physical, cognitive, emotional functioning, return to work)*		X			X	X	X
***Other**** (Resilience, quality of life, intervention changes and satisfaction, disability and functioning, health communication, and utilization of services*							
***Determination of costs during the study period***		X				X	X

*t1 *time point one, baseline assessment at approximately 3–4 months post injury; *t2 *time point two, follow-up at end of intervention at approximately 8–10 weeks after the baseline assessment; *t3 *time point three, follow-up at 3 months after intervention;* t4 *time point four, follow-up at 6 months after intervention

### Outcomes

#### Patient characteristics

The following sociodemographic variables will be registered at baseline: age, sex, marital status, educational level, living conditions, employment status, and annual income. Changes in living conditions, employment status and annual income will be recorded at the follow-ups.

#### Injury-related characteristics

Clinical and injury-related variables included comorbidities, diverse injury characteristics, and the trauma severity scores: the Abbreviated Injury Scale (AIS), Injury Severity Score (ISS), and NISS [22], length of hospitalization and medical treatment modalities, discharge place, and symptom burden along with screening of cognitive function in patients with suspected impaired cognitive function (MMS < 20). The trauma severity scores will be validated by data registered by certified AIS registrars in the hospitals’ trauma registries.

#### Outcome measures

When evaluating the effectiveness of complex interventions, it is recommended to use more than one outcome measure [25]. As the intervention aims to improve patients’ level of self-efficacy, the Trauma Coping Self-efficacy Scale [26] was chosen as the primary outcome with the 6-month follow-up (T4) being the endpoint. Secondary outcomes comprise domains such as symptom burden, physical and cognitive functioning, emotional distress, and return to work. Patients will nominate their target problem areas in their own words [27]. They will also be asked to nominate one self-selected outcome measure based on a presented list of domain-related measurements related to fatigue, sleep, pain, physical functioning, cognition, emotional functioning, and vocation. The rationale for including a patient-preferred outcome measure to ensure participant involvement and patient-centered outcome measurement. Their preferred outcome domain will be established as their self-selected secondary outcome at baseline. Additional outcomes include health-related quality of life, resilience, disability and global functioning, and patient impression of changes. All outcome measures are validated and presented in Table 2. The Trauma Coping Self-Efficacy Scale, the communication with physicians, and the Health Literacy Questionnaire were translated into Norwegian using the established translation procedures by a professional translation service (Forward translation; Expert panel discussion; Back-translation; Final version) [28]. Data for cost-effectiveness and cost-utility will be based on patient-reporting surveys of the type and frequency of received health care services. See Table 2 for an overview of the included outcome measures.

**Table 2:** Description of outcome measures utilized in the RCT

Domain:	Outcome measures:	Description:	Time point
**Primary outcome measures for all patients**			
Self-Efficacy	Trauma Coping Self-Efficacy (ref) [26]	Confidence in managing health problems and emotional functioning	T1–T4
**Secondary individual self-selected outcome measures by domain**			
Symptom burden	Rivermead Post-Concussion Symptom Questionnaire [29] Fatigue Severity Scale [30] Insomnia Severity Index [31] Brief Pain Inventory [32]	Symptom burden Fatigue Sleep disturbance Pain	T1–T4
Physical function	Short Physical Performance Battery [33] International Physical Activity Questionnaire Short Form [34]	Physical functioning level Overall physical activity and specific levels of intensity	T1–T4
Cognitive function	Cognitive Failures Questionnaire (CFQ) [35] Cognitive items Rivermead (memory, concentration, and mental speed) [29]	Self-reported cognitive failures Self-reported cognitive deficits	T1–T4
Emotional distress	Patient Health Questionnaire-9 [36] Generalised Anxiety Disorder-7 [37] Impact of Event Scale – Revised [38]	Depression Anxiety Post-traumatic stress	T1–T4
Return to work	Full-time/part-time (percentage); hours per week	Vocational participation	T1–T4
**Other outcome measurements for all patients**			
Resilience	Resilience Scale for Adults [39]	Level of Resilience	T1–T4
Health status (HRQL)	EuroQol five-dimensional questionnaire (EQ-5D) [40]	Health-related quality of life	T1–T4
Individual injury-related problem areas	Target Outcomes [27]	Individual target problem areas and their severity	T1–T4
Evaluation of changes and intervention satisfaction	Patient Global Impression of Change [41] Intervention satisfaction (intervention group only)	Level of satisfaction with intervention/ change	T2–T4 T2
Disability	WHO Disability Assessment Scale (WHODAS) [42]	Health and disability	T1–T4
General functioning	Glasgow Outcome Scale – Extended (GOSE) [43]	Global outcome	T1–T4
Communication	Communication with physicians [15] Health Literacy Questionnaire [44]	Communication with health care providers Capacity to obtain and understand health information	T1–T4
Health care utilization	Type and frequency of health care services received using a patient-reported survey. Health care services comprise secondary care services, primary care, specialist care, rehabilitation, and home- and community-based care, assistive devices, and informal care	Number of visits and type of health care services	T1–T4

### Process evaluation

Conducting a process evaluation as part of a randomized trial is recommended by the British Medical Research Council as it can add valuable knowledge about causal mechanisms, fidelity, and contextual factors that may influence both implementation of the intervention and variations in outcomes [25, 45]. In this study, the process evaluation will be based on information from the interventionists and participants using a mixed method approach by combining quantitative and qualitative data. This includes registration of participation rate, number of consultations apart from sessions and follow-ups, and direct and indirect time use of each consultation. Further, the completion of intervention due to protocol, variations, and reasons for non-compliance will be recorded. After the completion of the SEMPO, patients will be asked to evaluate the content and their satisfaction with the intervention using a semi-structured interview form.

### Interventions

#### Intervention group: self-management program (SEMPO)

The SEMPO program was developed by experienced clinicians and researchers and is based on well-known principles from the self-management concept and established rehabilitation strategies [11, 46]. Thus, SEMPO adheres to theories of self-efficacy as a mean of coping [11, 47]. The program aims to enhance self-efficacy by strengthening patients’ skills and confidence in managing the persisting consequences of the injury, as well as enhancing their health literacy and problem-solving competence. The program integrates components from evidence-based rehabilitation strategies within relevant functional domains [48, 49] to fit the symptom burden of the trauma population. User involvement was an essential part of the development of the intervention, and we collaborated with a user panel consisting of persons living with persistent injury-related consequences and representatives from the involved user organization, the National Association of the Traumatically Injured (LTN). In addition, the program has been adjusted after feedback from a test trial of the intervention (*n* = 5 persons), where patients were interviewed at the end of the testing regarding perceived usefulness and feedback on program content.

The SEMPO is delivered through 8 weekly 2.5-h sessions to groups consisting of 4–7 patients. A group format is chosen because social encouragement is considered a powerful means of increasing self-efficacy, thus SMS intervention delivered in groups is regarded as effective for behavior change, skills enhancement, and modeling [11]. Different components, such as psychoeducation, guided skills mastery, learning and practicing helpful compensatory strategies, and problem-solving techniques are integrated. In the weekly group sessions, topics with tailored psycho-educative content are presented and action plans reviewed, modified, and discussed in the group to help with problem-solving and address challenges. The manual will provide a fixed framework for the intervention, but action plans will be individualized and based on self-reported problems and individual challenges. In each session, patients will establish their own action plans containing achievable short-term weekly goals and related strategies. The program also includes a workbook, containing material from each session, self-monitoring schemes of weekly goal setting and goal attainment, as well as practice tasks between sessions (e.g., problem-solving of personal difficult tasks, strategy training of emotional worries). According to Bandura’s theory of self-efficacy [47], these ingredients may enhance a person’s self-confidence in managing trauma-related symptoms, problems in daily life activities, and participation. A tele-health version of the SEMPO intervention will be available for patients who prefer this or have to travel long distance to the hospital. In the tele-rehabilitation version, all participants will attend digitally, and the intervention will be delivered with the same content, frequency, and in-group mode. An overview of the intervention topics is displayed in Table 3.

**Table 3:** Overview of the intervention topics

No	Intervention topics
1	Introduction intervention/common injury consequences/action plans
2	Pain, use of medication, and nutrition
3	Active living, physical function, and exercise
4	Cognitive symptoms and compensatory techniques
5	Fatigue and sleep management
6	Managing psychological distress
7	Use of community resources and communication with health professionals
8	Summary and generalization of action plan for future problem areas

#### Criteria for discontinuing or modifying allocated interventions

The intervention will be delivered by a multidisciplinary team including medical doctors, psychologists, and physical therapists, all trained in self-management support strategies by Bridges Self-Management [50]. In any case of adverse effects of the intervention or in case of detected medical or psychological health problems in need of treatment, suitable actions will be discussed and ensured by the research team. A procedure for handling severe psychiatric conditions is included in the project, where the therapist will immediately consult with senior researchers who are specialist medical doctors and/or psychologists for further management.

#### Relevant concomitant care permitted or prohibited during the trial

Patients will not be withdrawn from any concurrent treatment during the trial.

#### Control group: treatment as usual

The control group will receive the usual health care and rehabilitation services provided in the municipality or other rehabilitation settings. Such services will potentially vary greatly depending on the patient needs and advice they receive at hospitals and services available in their municipalities, ranging from no services to regular contact with specialized or local rehabilitation teams. The services provided will be logged at baseline for all participants allowing comparison with the intervention group at all time-points in terms of content, extent, professionals involved, etc.

### Sample size

The sample size was estimated based on the primary outcome, the Trauma Coping Self-Efficacy Scale questionnaire [26]. The questionnaire is a 9-item scale answered on a scale ranging from 0 (not capable) to 7 (totally capable). A mean difference in the Trauma Coping Self-Efficacy Scale of 0.6 between baseline and follow-up between the groups is considered as a clinical relevant difference (ref). In order to detect a mean between-group difference of 0.6 with an estimated standard deviation of 1, equal allocation to both groups, 80% power, and a significance level of 5%, we will need to include a minimum of 45 patients in each group and a total of 90 participants [51]. With an assumed attrition rate of 20%, 110 patients will be included (i.e., 55 patients in each group). The sample size calculation was done based on the classical randomization arm (RA), and we aim to achieve the same power in the self-selection (SA) arm. However, the SA is considered an exploratory part of this study to evaluate the influence of patients’ treatment preferences. According to a previous longitudinal study at OUH, approximately 310 patients with trauma-related disability from 18 to 72 years of age may meet the inclusion criteria [52]. Based on previous studies, approximately 70–80% will be eligible for inclusion, resulting in 218–249 eligible patients per year.

### Recruitment

Patient recruitment is performed in collaboration with staff at the Department of Traumatology at OUH and the physicians allocated to the project through participation in the trauma report meetings and from lists of new hospitalized patients registered by the Department of Traumatology. One of the PhD candidates and a member of the project group (JNS + TH) will provide written and oral information to eligible patients, and those who agree to participate will be included upon discharge from the acute hospital stay or contacted by phone in cases of early discharge.

### Allocation/randomization

#### Sequence generation and allocation concealment mechanism

**Fig. 1:**
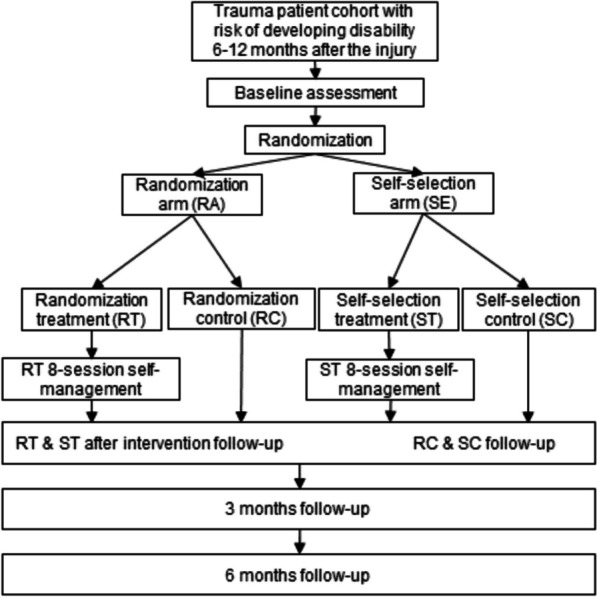
Participant timeline

The allocation sequence will be computer-generated by VieDoc using permuted blocks with varying block sizes. Patients will be randomized immediately after baseline assessment to RA or SA (see Fig. 1). Patients allocated to the RA will further be randomized to the intervention and control group, while patients randomized to the SA will self-select allocation to either intervention or control group.

#### Implementation

Eligible patients will be identified by project researchers (authors JSN and TH) in close collaboration with clinical staff at the Department of Traumatology at OUH.

#### Blinding/masking

Blinding of interventionists or patients will not be possible, but outcome assessors will be blinded for study allocation at follow-ups (T2–T4). The data analyst will be unaware of group allocation during the statistical analyses by assigning dummy codes to the study arms.

## Methods: data collection, management, and analysis

### Data collection methods

All patients will complete a baseline assessment before randomization procedures. The baseline assessments will be administered by the two PhD candidates (JNS and VMD). The patients can either answer the outcome questionnaires electronically or by pen and paper All outcome assessors will be trained in the administration of the data collection methods. A description of study outcome measures is displayed in Table 1. To ensure retention and complete follow-ups, the outcome assessors will be flexible with regards to scheduling the follow-up appointments. If patients wish to discontinue after the baseline assessment (T1), we will ask permission to use the baseline data in sensitivity analysis.

### Data management

Information about patients will be managed by healthcare professionals/project assistants adhering to Norwegian law on confidentiality [53]. All data material will be recorded with a participant ID, where only the local project group members will have access to the code list linking ID and patients. All data will be stored in VieDoc on a secured server at OUH, and individuals will not be identifiable in publications. The data will be stored securely in the VieDoc software and deleted 5 years after completion of the project.

The telehealth solution for self-chosen digitally delivered intervention will be provided by the Norwegian Health Net [54], a videoconference platform delivered by Pexip, Oslo, Norway. The platform is encrypted, and pin codes are used to access virtual meeting rooms, which are locked after start of meeting. Therapists will run the telehealth sessions using their work computers and a screen with an integrated speaker and camera optimized for videoconference. The videoconference solution is risk-assessed and approved for clinical use by the OUH.

### Statistical methods

Sociodemographic, clinical, and injury-related characteristics and outcome data will be presented using descriptive statistics. Independent sample *t*-test will be used for between-group mean comparisons for normally distributed continuous data, and Mann–Whitney *U*-tests for skewed data. For comparison of categorical data, chi-square tests or Fisher’s exact test will be used. To investigate the effectiveness of the intervention on self-efficacy, symptom burden and disability, and patient impression of change, mixed-effect models will be used to account for repeated measurements by patients. Time and time-by-treatment interaction will be used as fixed effects in these models. The linear mixed effects model will give estimated mean values for all time points (T1, T2, T3, and T4), changes from baseline, and between-group differences from baseline with 95% confidence intervals. The models will first be performed separately within the classical RCT and self-selected groups, and if reasonable, merged together. Possible confounders or mediators will be included when analyzing the self-selected group. To reduce the risk of dropout bias, the analyses will be based on an “intention to treat” principle by analyzing all patients in the group they were randomized to, regardless of whether they participated in or completed the intervention. A significance level of 5% will be used.

### Process evaluation analysis

We will conduct a process evaluation to investigate potential moderators of outcomes, such as intervention delivery, fidelity, and acceptability. The participation rate, numbers of consultations, the direct and indirect time related to each consultation, the kinds of problems presented, completion of intervention according to protocol, and any reasons for noncompliance will be assessed. Ten percent of intervention sessions will be overseen by a senior researcher aiming to evaluate treatment fidelity. Any need for adjustments with regard to adherence will be discussed in regular project meetings. We will also evaluate the experiences with the intervention for participants and therapists. The patients in the intervention group will rate intervention usefulness on a scale from 1 to 5 (not useful to extremely useful) and complete a semi-structured questionnaire about satisfaction with program content. After completion of the intervention, the therapist will rate the participant’s acceptability on a therapist checklist, by rating the degree of participation, interaction, and skill attainment on a scale from 0 to 3. The role of contextual factors will be explored in regression analysis, i.e., exploring external factors that may play into intervention effects.

### Additional analysis

#### Subgroup analysis

To acknowledge patient outcome preferences, we will also perform subgroup analysis based on the outcome preferences to identify which validated questionnaires can be considered as preferred patient-centered outcome measures in this or similar intervention studies, and whether the intervention might be more efficient on some problem domains than others.

#### Health economics analysis

Cost-effectiveness and cost-utility analyses of SEMPO compared to standard of care will be based on the effectiveness, measured by self-efficacy, symptom burden, global functioning, and quality-adjusted life years (QALYs). Use of healthcare and social support services (categories of service, quantities, and unit costs) collected during the study period, will be used to estimate total costs according to groups. In addition, vocational and educational rehabilitation, equipment and assistive technology, income, and informal care provided by family and significant others will be registered. We will estimate clinical effectiveness in QALYs using standardized conversion tools to convert health benefits into an index of HRQoL, as measured by EQ-5D [40]. Further, simulated cost consequences of the program will be considered from both a healthcare and societal perspectives [55]. Based on the estimated costs and QALYs, we will estimate the incremental cost-effectiveness ratio (ICER), defined as the differences in costs of the SEMPO and standard of care, relative to differences in QALYs of SEMPO and standard of care. Uncertainty will be presented by bootstrapping.

#### Managing non-adherence and missing data

Missing data will be handled according to the scoring manuals of the outcome measurements and by imputation. A sensitivity analysis will be performed to identify differences in patients’ characteristics between complete cases and dropouts.

### Monitoring

No data monitoring committee will be established for this trial because the trial does not include a pediatric population, has no potential to harm patients, will be performed in a short time frame, and is limited to a single center. Any substantial change to the study design affecting ethical or data protection issues will be reported to the Norwegian Regional Committee for Medical and Health Research Ethics (REK) and the Data Protection Office at OUH, respectively.

#### Adverse event reporting and harms

Any adverse events will be documented and reported by the project leader to the project owner, the Data Protection Office at OUH, and the ethical committee that have approved the study.

#### Auditing

No formal auditing is planned for this study. However, the Project Management Group will hold regular meetings to review the study process and implement necessary corrections as needed. Additionally, the extended project group, which includes all collaborators, will convene approximately twice a year to discuss the timeline and study progression. The steering committee will hold meetings twice every 6 months, to ensure progress and oversee conduct.

### Ethics and dissemination

#### Research ethics approval

The study has been approved by the Norwegian Regional Committee for Medical and Health Research Ethics (REK) (REK number 614625) and the Data Protection Officer at OUH. The study and all procedures will be conducted according to the declaration of Helsinki [56]. Information about participants will be handled by healthcare professionals adhering to Norwegian law on confidentiality. Informed consent will be obtained providing information about the right to withdraw from the project without giving any reason and without consequences for treatment access. Signed written informed consent forms will be collected from all participants by the PhD fellows. The involved user organization, the National Association of traumatically injured, LTN [57]will also take part in the monitoring and management of the research process.

#### Protocol amendments

As a preparation to the RCT, we will conduct a feasibility study, and necessary adjustments to the protocol will be discussed and decided in project group meetings and reported to the REK committee and the Data Protection Office at OUH. Changes in study procedures will be tracked and reported to investigators, trial registers, and health professionals working in the study.

#### Dissemination policy

The project aims to publish scientific papers in both national and international peer-reviewed journals. We expect to publish in high-quality scientific journals within the field of trauma rehabilitation due to the innovative aspects of the study and the important knowledge it contributes to enhancing post-acute treatment and rehabilitation following traumatic injuries. Findings will be presented at national and international scientific conferences, workshops and symposia, and to local and national clinical audiences. The study findings will be disseminated through the channels of the user organization. For study participants, we will create a lay abstract of the main findings. The dissemination reports and all papers will be written following the Consolidated Standards of Reporting Trials (CONSORT) to ensure transparency in reporting of the RCT [58]. In accordance with Norwegian Data Protection Laws, the data set will not be made available to the public. Anyone wishing to view the original data can do so by request to the corresponding author and physical presence at Oslo University Hospital, Norway.

## Discussion

This self-management intervention study targets patients with moderate to severe physical trauma in the sub-acute phase and patients at risk of long-term symptom burden and injury-related disabilities. The innovative and newly designed self-management program; SEMPO is a complex intervention and entails ingredients aiming to enhance patients’ self-efficiency in managing difficulties they experience in daily life in the sub-acute phase following injury. The program includes therapeutic ingredients such as psychoeducation, action planning, training in problem-solving, and peer-sharing experience in managing injury-related consequences. The program focuses on establishing and reviewing personal action plans, facilitating individual skills enhancement, and sharing experiences to enhance social encouragement. To our knowledge, this is the first study to investigate the effect of a self-management program tailored to patients with moderate to severe physical trauma in the sub-acute phase of recovery. It is also the first study to bring the self-management concept into a structured and manualized group-based intervention in the trauma population. The intensity of the program, with eight weekly sessions, allows thorough training in goal setting and adaptive management of difficulties. The intervention is in line with person-centered treatment.

The inclusion of a self-selection arm in a pragmatic RCT is an innovative way to incorporate the influence patients’ preferences have on the effectiveness of the intervention. This approach brings the experimental design for clinical interventions closer to real-world scenarios and aligns with the notion of shared decision-making. The self-selection arm is an explorative part of the study, while the classic randomization arm (patients allocated by randomization to either intervention or control group), will follow a more traditional rigorous experimental design.

### Limitations

This is a pragmatic clinical trial and blinding of patients and therapists is not feasible. However, outcome assessors will be blinded to group allocation. The treatment as usual may potentially vary based on demographic factors, as well as injury-related factors. Treatment as usual is not standardized in the study, but it does reflect the real differences in treatment access and availability. Another possible limitation is the risk of non-adherence and dropouts at follow-up and potential differential drop-out according to group allocation and/or etiology. To facilitate study adherence and prevent drop-outs, the research team will be well-trained, conduct outreach and offer flexibility regarding timing and intervention delivery (e.g., self-selection of face-to-face mode or telehealth delivery).

### Trial status

Protocol version 1.0. Recruitment for the RCT began in January 2024 and will continue until target sample size has been reached, estimated by the end of 2026.

## Supplementary Information

Additional file 1. Informed consent material

Additional file 2. Approval from the Norwegian Regional Committee for Medical and Health Research Ethics (REK)

Additional file 3. Proof of funding from the South-Eastern Norway Regional Health Authority

Additional file 4.

## Data Availability

In accordance with Norwegian Data Protection Laws, the data set will not be made available to the public. Anyone wishing to view the original data can do so by request to the corresponding author and physical presence at Oslo University Hospital, Norway.
