# Supplementary material for: Self-management support program delivered in the sub-acute phase after traumatic injury—study protocol for a pragmatic randomized controlled trial
Source: Trials. 2024 Sep 30;25:639. doi: 10.1186/s13063-024-08492-0 (PMC11441131; doi:10.1186/s13063-024-08492-0)
Supplement: Supplementary file 2 — Additional file 2. Approval from the Norwegian Regional Committee for Medical and Health Research Ethics (REK) [file 13063_2024_8492_MOESM2_ESM.pdf]

Oslo universitetssykehus

Forespørsel om deltakelse i en utprøvingsstudie

-Utvikling av selvmestringsprogram for traumatiske skader i sub-akutt fase

Bakgrunn
Dette er en forespørsel til deg om å delta i en utprøvingsstudie som retter seg mot personer som har gjennomgått en traumatisk skade. Studien har til hensikt å prøve ut et selvmestringsprogram som utvikles i samarbeid mellom Oslo Universitetssykehus, Ullevål og Sunnaas sykehus HF.

Hva innebærer studien?
Deltakelse innebærer at du vil motta et gruppebasert selvmestringsprogram som har som formål å styrke helsekompetanse og mestringstro. Programmet vil gå over fire uker med to ukentlige treff à cirka 2,5 timer. Gruppen vil bestå av 5 - 7 deltakere. Intervensjonen blir gjennomført av tverrfaglig personell. Innholdet i programmet vil inkludere øvelser for å øke mestringstro, trening i strategier og problemløsning, arbeid med små individuelle mål knyttet til egen hverdag, samt erfaringsutveksling mellom deltakerne. I tillegg gis det informasjon om ulike symptomer og utfordringer som mange med traumatisk skade opplever som konsekvenser etter skaden. Programmet inneholder sesjoner med følgende temaer 1) Vanlige skadekonsekvenser, introduksjon av individuelle ukentlige mål; 2) Smerter, kosthold og bruk av medisiner; 3) Aktiv livsstil, fysisk funksjon og trening; 4) Kognitive symptomer og kompenserende strategier; 5) Mestring av fatigue og søvnvansker; 6) Mestring av psykiske plager og stress; 7) Bruk av helsetjenester og kommunikasjon med helsepersonell; 8) Oppsummering og videre bruk av målarbeid i din hverdag. Opplevd nytteverdi av selvmestringsprogrammet vil bli kartlagt etter intervensjonen. Etter fullført deltakelse i programmet vil vi derfor gjerne intervjue deg for å høre om dine erfaringer og utbytte for å evaluere selvmestringsprogrammet. Dine innspill med tanke på omfang, form og innhold vil være viktige bidrag til at vi som fagfolk lager et program med god nytteverdi for pasienter som har vært utsatt for en fysisk skade.

Mulige fordeler og ulemper
Deltakelse i studien innebærer ingen helserisiko. Deltakelse vil ikke medføre ulemper utover økt tidsbruk ved gjennomføring av programmet. Det vil kunne være til fordel for pasienter med traumatisk skade at det utvikles et program som kan styrke deres helsekompetanse og mestringsevne. Dette kan potensielt senere benyttes til å forbedre helsetilbudet til pasientgruppen som helhet og hjelpe andre med liknende problemer.

Hva skjer med prøvene og informasjonen om deg?
Alle opplysningene vil bli behandlet uten fødselsnummer eller andre direkte identifiserbare opplysninger, men navn vil bli registrert. En kode knytter deg til dine opplysninger gjennom en navneliste som vil bli oppbevart i låst arkivskap. Det er kun autorisert personell knyttet til utprøvingsstudien som kan finne tilbake til deg. Du har rett til innsyn i hvilke opplysninger som er registrert om deg og rett til å få korrigert eventuelle feil i de opplysningene som er registrert. Det vil ikke være mulig å identifisere deg i resultatene av studien når disse publiseres. Opplysningene blir senest slettet 31.12.2024.

Frivillig deltakelse
Det er frivillig å delta i studien. Dersom du ikke ønsker å delta, trenger du ikke å oppgi noen grunn, og det får ingen konsekvenser for videre behandling ved sykehuset. Dersom du ønsker å delta, undertegner du samtykkeerklæringen på siste side. Du kan når som helst og uten å oppgi noen grunn trekke ditt samtykke. Dette vil ikke få konsekvenser for din videre behandling. Dersom du trekker deg fra studien, kan du kreve å få slettet innsamlede opplysninger, med mindre opplysningene allerede er anonymisert og inngått i analyser.

Forsikring
Du er forsikret etter pasientskadeloven under deltagelsen i studien.

Godkjenning
Personvernombudet har vurdert prosjektet, og har gitt forhåndsgodkjenning (Ref. nr 23/04659).

Oslo universitetssykehus

Etter ny personopplysningslov har behandlingsansvarlig OUS og prosjektledere (Solveig L. Hauger og Nada Andelic) et selvstendig ansvar for å sikre at behandlingen av dine opplysninger har et lovlig grunnlag. Dette prosjektet har rettslig grunnlag i EUs personvernforordning artikkel 6 nr. 1a og artikkel 9 nr. 2a og ditt samtykke. Du har rett til å klage på behandlingen av dine opplysninger til Datatilsynet.

Kontaktopplysninger

Dersom du har spørsmål om prosjektet kan du kontakte:

- Prosjektleder ved OUS overlege Nada Andelic Tlf. 918 17 910, e-post nadand@ous-hf.no,
- Prosjektleder ved Sunnaas sykehus Solveig L. Hauger Tlf. 91310358, e-post solveig.laegreidhauger@sunnaas.no
- Prosjektmedarbeider Mari S. Rasmussen Tlf. 99797575, e-post masras@ous-hf.no

Personvernombud ved institusjonen er Tor Åsmund Martinsen [personvern@oslo-universitetssykehus.no].

Samtykke for deltakelse i studien

Jeg er villig til å delta i studien

(Signert av prosjektdeltaker, dato)

Bekreftelse på at informasjon er gitt deltakeren i studien

Jeg bekrefter å ha gitt informasjon om studien

(Signert, rolle i studien, dato)
