# Supplementary material for: Self-management support program delivered in the sub-acute phase after traumatic injury—study protocol for a pragmatic randomized controlled trial
Source: Trials. 2024 Sep 30;25:639. doi: 10.1186/s13063-024-08492-0 (PMC11441131; doi:10.1186/s13063-024-08492-0)
Supplement: Supplementary file 3 — Additional file 3. Proof of funding from the South-Eastern Norway Regional Health Authority [file 13063_2024_8492_MOESM3_ESM.pdf]

Oslo University Hospital

Oslo University Hospital

Postal address:
Trondheimsveien 235
NO-0514 Oslo
Norway

Switchboard:
+47 915 02770

Org.number:
NO 993 467 049 MVA

www.oslo-universitetssykehus.no

To whom it may concern

Date: 22nd of April 2024

Proof of funding

This is to confirm that Nada Hadzic-Andelic is employed at the Oslo University Hospital HF, and has received a research grant from the South-Eastern Norway Regional Health Authority (HSØ), amounting to NOK 8 775 000,- from 1st of January 2023 and for the duration of three years. The project title is; "Managing symptoms and disability in the sub-acute phase after traumatic injury - A pragmatic randomized controlled trial of a self-management support program"

Please do not hesitate to contact me should there be any questions or further information needed.

Administrativt ansvarlig/Prosjektadministrator/Project Administrator/Authorized Representative

Martin Sending
Head of department
Administrative Research Support
Research Support Unit - Oslo Hospital Service
Oslo University Hospital
